# Preparation of Protein A Membranes Using Propargyl Methacrylate-Based Copolymers and Copper-Catalyzed Alkyne–Azide Click Chemistry

**DOI:** 10.3390/polym16020239

**Published:** 2024-01-15

**Authors:** Joshua Osuofa, Scott M. Husson

**Affiliations:** Department of Chemical and Biomolecular Engineering, Clemson University, Clemson, SC 29634, USA; josuofa@g.clemson.edu

**Keywords:** AGET ATRP, affinity chromatography, antibody purification, surface-initiated polymerization

## Abstract

The development of convective technologies for antibody purification is of interest to the bioprocessing industries. This study developed a Protein A membrane using a combination of graft polymerization and copper(I)-catalyzed alkyne–azide click chemistry. Regenerated cellulose supports were functionalized via surface-initiated copolymerization of propargyl methacrylate (PgMA) and poly(ethylene glycol) methyl ether methacrylate (PEGMEMA_300_), followed by a reaction with azide-functionalized Protein A ligand. The polymer-modified membranes were characterized using attenuated total reflectance Fourier-transform infrared spectroscopy (ATR-FTIR), gravimetric analysis, and permeability measurements. Copolymer composition was determined using the Mayo–Lewis equation. Membranes clicked with azide-conjugated Protein A were evaluated by measuring static and dynamic binding (DBC_10_) capacities for human immunoglobulin G (hIgG). Copolymer composition and degree of grafting were found to affect maximum static binding capacities, with values ranging from 5 to 16 mg/mL. DBC_10_ values did not vary with flow rate, as expected of membrane adsorbers.

## 1. Introduction

Protein A affinity chromatography is a unit operation in the downstream purification of monoclonal antibodies and other Fc-based proteins. The selectivity of Protein A chromatography results in nearly 99% removal of such impurities as host cell proteins (HCPs), host cell DNA (HcDNA), and host cell viruses in a single step. Although alternatives to Protein A chromatography are being studied, such as chromatography with synthetic ligands, affinity precipitation, cation exchange, and multimodal chromatography, these alternative capture-step purification technologies do not match the selectivity of Protein A chromatography [1,2]. Additionally, the bioprocessing industry is slow to adopt new capture-step purification technologies. So there is a continued incentive to improve Protein A chromatography media.

Improvements are needed to overcome several challenges in Protein A chromatography. Packed-bed Protein A chromatography using resin particles suffers from diffusional mass-transfer limitations that cause an inverse relationship between flow rate and dynamic binding capacity. Additionally, the volume of Protein A resin columns, at scale, cannot be increased without significant packing issues that cause channeling and resin particle degradation that can cause high pressures and loss of media. Membrane chromatography can overcome these challenges. Because protein transport to binding sites is driven by convective flow rather than diffusion, membrane chromatography yields higher throughput and flow-rate-independent binding capacity [3,4,5,6].

Previous Protein A membranes have been prepared using several immobilization chemistries targeting the amine groups of Protein A, including activation of supports with EDC (1-ethyl-3-(3-dimethylaminopropyl)carbodiimide)/NHS (N-hydroxysuccinimide) [7,8], cyanogen bromide [9], glutaraldehyde [10], disuccinimidyl carbonate [10], aldehyde [11,12], and epoxide groups [13]. Among these publications, only one utilized a graft polymerization approach. Ma et al. [7] prepared Protein A membranes using cerium (IV) ion-induced graft polymerization of carboxylic acid-containing polymers from electrospun PES supports and subsequently immobilized Protein A using EDC/NHS chemistry. However, Protein A was immobilized under one condition, and no attention was paid to the effects of polymer composition and degree of grafting on performance or the thermodynamic behavior of protein adsorption on the synthesized Protein A membrane.

In this study, we utilized the copper(I)-catalyzed alkyne–azide click reaction chemistry, known for its bio-orthogonality, high specificity, irreversibility, fidelity (i.e., no side products), rapid rate of reaction [14,15,16], and quantitatively high yields (>90%) [17], to synthesize Protein A membranes. Azide-functionalized Protein A ligands were immobilized on a macroporous membrane that was first modified by grafting copolymers of the alkyne monomer propargyle methacrylate (PgMA) and the spacer monomer poly(ethylene glycol) methyl ether methacrylate (PEGMEMA_300_) at varying PgMA-*co*-PEGMEMA_300_ compositions using activators generated by electron transfer atom transfer radical polymerization (AGET ATRP). Membranes were analyzed using gravimetric analysis, water permeability measurements, and attenuated total reflectance Fourier-transform infrared (ATR-FTIR) spectroscopy. Antibody binding performance was assessed by measuring the dynamic and static binding capacities of human immunoglobulin G. To our knowledge, this is the first publication investigating the effect of polymerization conditions on the performance of Protein A affinity membranes.

## 2. Materials and Methods

### 2.1. Materials

The following materials were purchased from MilliporeSigma (St. Louis, MO, USA): 3-azido-1-propanol (>96%), α-bromoisobutyryl bromide (2-Bib, 98%), acetonitrile (HPLC grade, >99.9%), alpha-cyano-4-hydroxycinnamic acid (α-CHCA, 99%), ascorbic acid (99%), copper (II) bromide (CuBr_2_, 99%), dimethyl sulfoxide (DMSO, anhydrous, >99.9%), formic acid (reagent grade, >95%), N,N,N′,N″,N″-pentamethyldiethylenetriamine (PMDETA), phosphate-buffered saline (PBS, powder, Product number P3813), poly(ethylene glycol) methyl ether methacrylate (PEGMEMA, M_n_ = 300 g/mol), sodium bicarbonate (>99.5%, anhydrous), tetrahydrofuran (THF, anhydrous, >99.9%, inhibitor-free), triethylamine (TEA, >99.5%), tris(2-pyridylmethyl)amine (TPMA, 98%), tris(3-hydroxypropyltriazolylmethylamine) (THPTA, 95%), and Whatman regenerated cellulose membrane filters (RC-60, 47 mm diameter, nominal pore size of 1 μm).

Polyclonal immunoglobulin G from human plasma (hIgG, product number 340-21, >95%) was purchased from Lee Biosolutions (Maryland Heights, MO, USA). Propargyl methacrylate (PgMA, 98%) was purchased from Alfa Aesar (Haverhill, MA, USA). Native recombinant staphylococcal Protein A ligand (rSPA, Product number 10-2001-0M) was purchased from Repligen (Waltam, MA, USA). Azidobutyric acid N-hydroxysuccinimide (NHS) ester (catalog number 63720, 95%) was purchased from Lumiprobe Life Science Solutions (Hunt Valley, MD, USA).

### 2.2. Preparation of Azide-Functionalized Protein A

#### 2.2.1. Reaction of Protein A with Azidobutyric Acid NHS Ester

The conjugation reaction was carried out using a 15-to-1 molar ratio of azidobutyric acid NHS ester to Protein A. Protein A stock solution was supplied by Repligen at 50 mg/mL in a sodium chloride buffer solution. The Protein A reaction solution was prepared by adding the Protein A stock solution (1.3 mL) to a sodium bicarbonate solution (138 mM, 4.7 mL) at pH 8.4. A solution of azidobutyric acid NHS ester (40 mM) was prepared in anhydrous DMSO. The conjugation reaction was initiated by adding the azidobutyric acid NHS ester solution (521 µL) dropwise to the Protein A reaction solution while mixing it at 60 rpm. After that, the reaction proceeded for 12 h at room temperature. The final solution was 10 mg/mL conjugated Protein A.

#### 2.2.2. Matrix-Assisted Laser Desorption Ionization-Time of Flight (MALDI-TOF) Mass Spectrometry

MALDI-TOF spectrometry was performed to determine the degree of conjugation. The matrix was prepared with 20 mg/mL α-CHCA in a 70:30 (*v*/*v*) mixture of acetonitrile and 5 vol% formic acid (aq.). The plating procedure was as follows. First, the matrix solution (1 µL) was plated and allowed to dry. Next, the conjugated Protein A (1 µL) solution was plated. Finally, the matrix (1.5 µL) was added. Samples were allowed to air dry for 30 min before analysis. All samples were analyzed using a Bruker Microflex^TM^ LRF MALDI-TOF (Billerica, MA, USA) in linear mode (105 cm flight path). The laser level was between 20 and 70%, the detector gain was 2827 V, and the analysis used a positive ion spectrum. Reported MW/charge (M/Z) values are the average of the peak of two different measurements, with the error bars representing the standard deviation.

### 2.3. Membrane Preparation

**Scheme 1:**
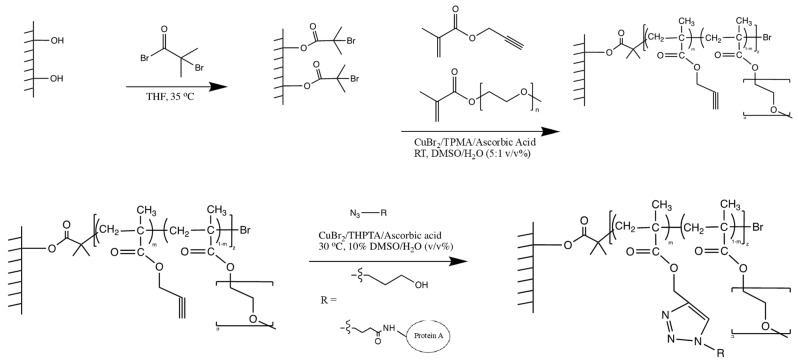
Preparation strategy for Protein A membranes utilizing surface-initiated AGET ATRP and click chemistry.

Scheme 1 illustrates the synthetic pathway to prepare Protein A membranes. First, the initiator molecules were anchored to the surfaces of the membrane pores. Then, AGET ATRP was used to graft a copolymer of PgMA and PEGMEMA_300_ from the initiation sites. Azide-functionalized Protein A or azido-propanol was clicked onto the alkyne groups of the grafted copolymer. Details for each step are provided in the sections that follow.

#### 2.3.1. Initiator Functionalization of Regenerated Cellulose Membranes

Functionalization was performed using an esterification reaction that converts the hydroxyl groups of the cellulose membrane to α-bromoester initiator groups. Hydrobromic acid is released as a byproduct that is neutralized by TEA.

RC-60 membranes were rinsed by immersing them in anhydrous THF (50 mL) for 10 min before initiator functionalization. After rinsing, the membranes were immersed in the functionalization solution comprising 2-Bib (18 mM, 111 μL), TEA (18 mM, 125 μL), and anhydrous THF (50 mL). The 2-Bib and TEA volumes were doubled for membranes initiated with 36 mM 2-Bib. The reaction was carried out at 35 °C for 2 h in a low oxygen (<1 ppm) UNIlab glove box (MBraun, Stratham, NH, USA). After the reaction, membranes were rinsed and stored in anhydrous THF.

#### 2.3.2. AGET ATRP of PEGMEMA_300_ and PgMA

RC-60 membranes functionalized with 2-Bib were modified further via surface-initiated AGET ATRP of PgMA and PEGMEMA_300_. Before polymerization, PgMA and PEGMEMA_300_ were passed through a column of aluminum oxide to remove the inhibitor. The monomers were used at pre-determined molar percentages ranging from 0 to 100% PgMA. At each composition, the total monomer concentration was set to 1.6 M. In this example procedure, 30 mol% PgMA was used. The solvent was a 5:1 (*v*/*v*) mixture of DMSO (3.77 mL) and water (0.75 mL). Components of the catalyst complex, CuBr_2_ (22.5 mM, 30 mg) and TPMA (45 mM, 78.5 mg), were added to the solvent, and the solution was sonicated until a homogenous cyan color was formed. PgMA (0.480 M, 0.363 mL) and PEGMEMA_300_ (1.120 M, 1.920 mL) were added to the solution. Two 25 mm diameter RC-60 membrane coupons were added to the glass vial containing the reaction solution. The vial was sealed with a crimp cap and moved into the glove box. An ascorbic acid stock solution (0.1 M) was prepared by adding ascorbic acid (53 mg) to deionized water (3 mL). The ascorbic acid stock solution (135 µL, representing a 1:10 molar ratio relative to CuBr_2_) was added to the reaction solution in the glove box to reduce CuBr_2_ to CuBr. The polymerization was carried out for 5 h at room temperature.

#### 2.3.3. Click Reaction onto PgMA-co-PEGMEMA_300_ Modified Membranes

Two molecules were used for the click reaction onto PgMA-*co*-PEGMEMA_300_-modified membranes: azido-propanol and azide-functionalized Protein A. Azido-propanol was used to demonstrate the feasibility of the click reaction protocol. The click reaction occurred in 1:9 DMSO/H_2_O (*v*/*v*). Final solution concentrations are given in parentheses.

Azido-propanol (60 µL, 120 mM) was added to deionized water (5.3 mL) in a 30 mL glass vial. The catalyst complex comprising CuBr_2_ (16 mg, 11.7 mM) and THPTA (30.5 mg, 11.7 mM) was prepared in DMSO (0.610 mL) and then added to the azido-propanol solution. A stock solution of 1 M sodium ascorbate was prepared in deionized water. Three PgMA-*co*-PEGMEMA_300_-functionalized membranes of 10 mm diameter were added to the solution, the vial was sealed with a crimp cap, and sodium ascorbate stock solution (0.210 mL, representing a 3:1 molar ratio relative to CuBr_2_) was injected into the solution using a syringe to reduce CuBr_2_ to CuBr. The vial was placed into a glovebox at room temperature for 12 h. After the click reaction, membranes were rinsed sequentially in 100 mM citric acid at pH 3, 100 mM sodium hydroxide, and 1 M sodium chloride in an ice bath. Membranes were stored in a 1x PBS solution in a refrigerator until further use.

The same procedure was followed for a reaction with azide-functionalized Protein A, but the protein concentration was 7 mg/mL and one 18 mm diameter PgMA-*co*-PEGMEMA_300_ functionalized membrane was put into the click reaction solution.

### 2.4. Membrane Characterization

#### 2.4.1. Attenuated Total Reflectance Fourier-Transform Infrared (ATR-FTIR) Spectroscopy

ATR-FTIR spectroscopy was used to analyze the surface chemistry of RC-60 membranes before and after initiator functionalization, AGET ATRP modification, and the Protein A click reaction. A Perkin Elmer Spectrum Two FTIR was used for analysis of all samples. The instrument had a Universal ATR accessory and a single reflection diamond. Each spectrum was obtained using 32 scans at a resolution of 4 cm^−1^. Background correction, baseline correction, ATR-FTIR correction, and peak analysis were performed using PerkinElmer Spectrum 10 software.

#### 2.4.2. Water Permeability Measurements

Pure water permeability measurements were performed using a Millipore stirred ultrafiltration cell (model 8050). Each membrane was placed in the ultrafiltration cell, and a transmembrane pressure of 2 bar was applied using an air gas cylinder. Water flowed through the membrane for 30 s. Permeability was calculated as the quotient of the mass of water permeated and the multiplication product of the surface area, flow time, and transmembrane pressure. Experiments were repeated in triplicate. Reported values are the averages, with error bars representing the standard deviation.

#### 2.4.3. Gravimetric Analysis

The degree of ATRP modification was determined gravimetrically as a function of the molar concentration of PgMA in the ATRP solution. At least three membranes of 10 mm diameter were modified for each ATRP formulation. Pre- and post-ATRP modification, the membranes were dried in a desiccator for at least 7 d before the mass measurements were taken to minimize any influence that humidity fluctuations may have on mass measurements. The degree of grafting (DG) was calculated by Equations (1) and (2), where *w*_0_, *w*_1_, and *w*_2_ are masses of unmodified, initiator-activated, and polymer-grafted membranes. *DG_init_* is the degree of grafting for initiator functionalized membranes with respect to unmodified membranes, and *DG_poly_* is the degree of grafting for PgMA-*co*-PEGMEMA_300_-modified membranes with respect to initiator functionalized membranes. All reported values are an average of at least three measurements.

(1) $${DG}_{init}\left(\%\right)=\frac{\left(w_{1}-w_{0}\right)100}{w_{0}}$$

(2) $${DG}_{poly}\left(\%\right)=\frac{\left(w_{2}-w_{1}\right)100}{w_{1}}$$

### 2.5. Performance of Protein A Membranes

#### 2.5.1. Static Binding Capacity (SBC)

The SBCs of Protein A membranes were measured using hIgG solutions prepared at initial concentrations ranging from 0.1 to 5 mg/mL in 1x PBS buffer. Two dried Protein A membranes were placed into a 20 mL glass vial containing 5 mL hIgG solution. The vial was put in a shaker bath at 22 °C and 120 rpm for 24 h. The equilibrium hIgG concentration was measured with a Nanodrop^TM^ One Microvolume UV-Vis Spectrophotometer (ThermoFisher Scientific, Waltham, MA, USA) at 280 nm. A calibration curve was created to relate hIgG concentration in solution to Nanodrop^TM^ output values. A mass balance using the initial and equilibrium hIgG concentrations and the volume of the membranes was used to calculate the SBC from Equation (3). *q* is the SBC (mg protein/mL column volume), *C_o_* is the initial concentration (mg protein/mL solution), *C_eq_* is the equilibrium protein concentration (mg protein/mL solution), *V_col_* is the membrane volume (mL), and *V_sol_* is the volume of protein solution (mL). *V_col_* was calculated by multiplying the membrane area by the thickness measured by a micrometer. Reported SBC values represent an average of at least two runs at each condition.

(3) $$q=\frac{{\left(C\right.}_{o}\;-\;C_{eq})V_{sol}}{V_{col}},$$

Thermodynamic parameters were determined by fitting SBC values using the Langmuir isotherm model (Equation (4)). *q_max_* is the maximum binding capacity (mg protein/mL membrane), and *K_d_* is the apparent Langmuir dissociation equilibrium constant (mg/mL). Fitted parameters (*q_max_*_,_ *K_d_*) and their standard errors were determined using the *fitnlm* command in MATLAB software.

(4) $$q=\frac{q_{max}C_{eq}}{K_{d}+C_{eq}},$$

#### 2.5.2. Dynamic Binding Capacity (DBC_10_)

DBC_10_ measurements were performed using an AKTA Purifier 100 with Unicorn software (5.31) from Cytiva (Marlborough, MA, USA). hIgG solutions for all measurements were prepared by dissolving the protein in the loading buffer (1x PBS, pH 7.4) and filtering using a 0.2 µm cellulose acetate filter before use. DBC_10_ values (mg protein/mL membrane volume) were calculated using Equation (5). *V_break_* is the effluent volume (mL) at 10% breakthrough (i.e., where effluent concentration equals 10% of the initial concentration), and *V_dead_* is the dead volume of the system (mL). DBC_10_ values reported represent an average of at least three runs, with error bars being the standard deviation.

(5) $${DBC}_{10}=\frac{{\left(V\right.}_{break}-V_{dead})C_{o}}{V_{col}},$$

## 3. Results and Discussion

### 3.1. Preparation of Azide-Functionalized Protein A

Protein A contains 55 lysine residues and one N-terminal. It is unclear how many of these are surface residues, but 30 are in the X domain, originally where the protein was covalently bound to the staphylococcal bacteria cell wall [18]. Azide functionality was introduced into the Protein A structure via NHS ester chemistry involving these lysine groups. At pH values greater than 8.0, primary amines in the lysine residues of Protein A performed a nucleophilic attack on the carbonyl carbon of the azidobutyric acid NHS ester. The conjugation reaction was carried out using a 15-to-1 molar ratio of azidobutyric acid NHS ester to Protein A to promote conjugation.

**Figure 1:**
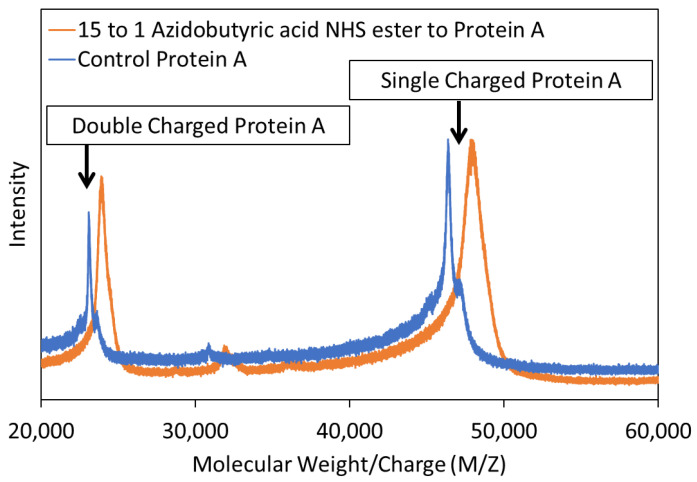
MALDI-TOF spectrometry spectra of azide-conjugated Protein A (orange) and control Protein A (blue). Protein A was conjugated with azidobutyric acid NHS ester at a molar ratio of 15 to 1 in solution.

The results of MALDI-TOF spectrometry analysis illustrated in Figure 1 and summarized in Table 1 indicate an increase in molecular weight of conjugated Protein A relative to control Protein A. Each conjugated azidobutyric acid NHS ester adds 111 g/mol in molecular weight. The difference in mass between conjugated and native Protein A from both the singly charged and doubly charged peaks correspond to additions of between 13 and 14 azide moieties on each Protein A molecule. The high conjugation rate may be due to the well-known rod-like extended shape of Protein A, which allows access to many surface lysine groups [19].

### 3.2. Characterization of ATRP Modified Membranes

#### 3.2.1. ATR-FTIR of ATRP Modified Membranes

**Figure 2:**
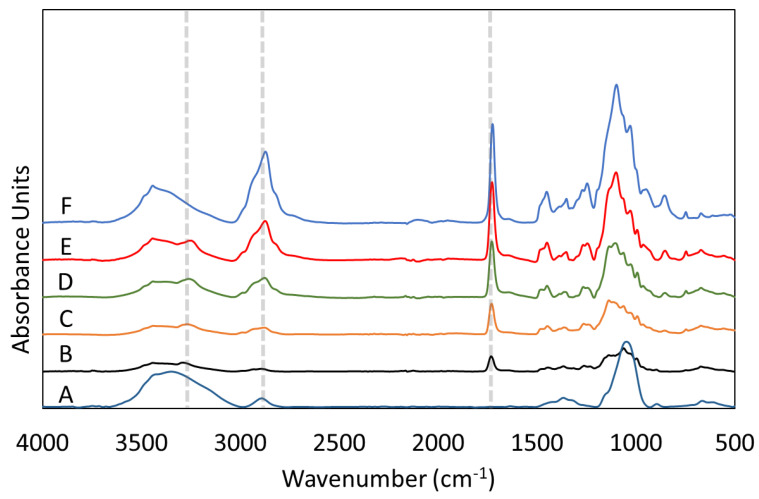
ATR-FTIR of regenerated cellulose membranes after modification using surface-initiated AGET ATRP of PgMA-*co*-PEGMEMA_300_ copolymer: (**A**) unmodified membrane, (**B**) 100% PgMA-*co*-0% PEGMEMA_300_, (**C**) 70% PgMA-*co*-30% PEGMEMA_300_, (**D**) 50% PgMA-*co*-50% PEGMEMA_300_, (**E**) 30% PgMA-*co*-70% PEGMEMA_300_, (**F**) 0% PgMA-*co*-100% PEGMEMA_300_. Percentages represent the initial molar percentages of each monomer in solution during AGET ATRP. Gray dashed lines indicate the prominent distinguishing peaks for different copolymer compositions.

Figure 2 shows the resulting ATR-FTIR spectra of membranes modified using the grafted copolymers at varying compositions. Regardless of PgMA percentage, all spectra contain a peak at 1720 cm^−1^, which is assigned to the carbonyl moiety. Since both PgMA and PEGMEMA_300_ have this functional group, it is present in all cases and supports successful surface-initiated polymerization. The differences in peak heights at 1720 cm^−1^ are due to different polymerization rates of the two monomers. The prominent distinguishing peaks for different copolymer compositions can be found by comparing the samples polymerized with PgMA and those polymerized without it. Samples containing PgMA (spectra B-E) show a peak at 3245 cm^−1^ that can be assigned to the alkyne moiety, while spectrum F for the 100% PEGMEMA_300_ sample does not.

Additionally, for the 100% PEGMEMA_300_ sample, there is a prominent peak near 2874 cm^−1^ indicative of C-H stretching in PEG [20]. For PEGMEMA_300_, similar peaks have been reported in previous papers [21,22]. As the molar percentage of PEGMEMA_300_ increases from 0 to 100% in the formulation, the PEG C-H stretching peak at 2874 cm^−1^ increases. The analysis of peaks at 1720 cm^−1^ and 2874 cm^−1^ supports successful surface-initiated AGET ATRP for all cases.

#### 3.2.2. Gravimetric Analysis of ATRP Modified Membranes

**Figure 3:**
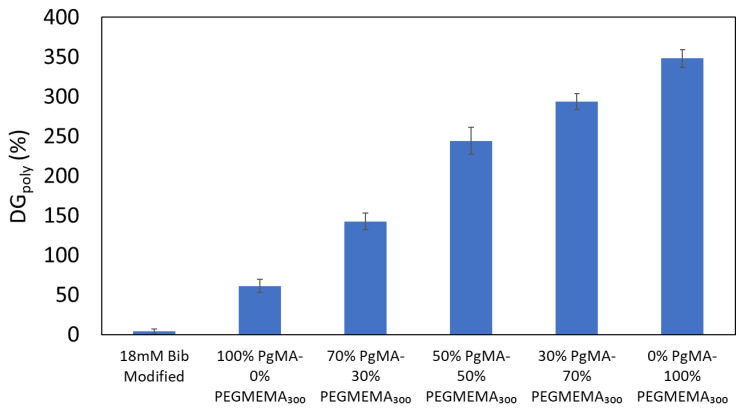
Degree of grafting of PgMA-*co*-PEGMEMA_300_ modified membranes. Error bars represent the standard deviation of at least three measurements.

Copolymer-modified membranes were stored in a desiccator for 7 d before mass measurements. Before mass measurement, desiccation was required to remove unbound water from the membrane. The DG_poly_ was calculated relative to the initiator-modified membrane. The initiator-modified membrane (labeled 18 mM Bib) showed a 4% increase in mass relative to the unmodified membrane, while the polymer-modified membranes showed much more significant increases. Figure 3 shows that the DG_poly_ increased with an increasing percentage of PEGMEMA_300_ in the copolymer solution. The case with 100% PgMA in solution resulted in 61.3 ± 8.5% DG_poly_, while the 100% PEGMEMA_300_ resulted in 348.1 ± 11.1% DG_poly_. Based on the homo-polymerization cases, the ratio of moles of grafted PEGMEMA_300_ to grafted PgMA was 2.35 to 1, indicating that the rate of polymerization for PEGMEMA_300_ was much faster than the rate of polymerization for PgMA. Membrane thickness (Figure S1 in Supplementary Materials) also increased significantly due to polymerization, and the increase in thickness followed the same trend as the mass increase.

Using the rates of homo-polymerization from the gravimetric analysis, the Mayo–Lewis equation (Equation (6)) was used to approximate the molar ratio of PgMA to PEGMEMA_300_ in grafted copolymers. The Mayo–Lewis equation assumes that the molar concentration of the respective monomers in the solution remains constant. This assumption is valid for surface-initiated polymerization from macroporous membranes because of the low conversion (0.26% for 100% PEGMEMA_300_, 0.11% for 100% PgMA) of monomers in solution to the grafted polymer. In Equation (6), the relative rate of polymerization for PgMA (*r*_1_) was set to 1, the relative rate of polymerization for PEGMEMA_300_ (*r*_2_) was 2.35, and the solution concentrations for PgMA and PEGMEMA_300_ are denoted as [PgMA] and [PEGMEMA_300_].

(6) $${\left(\frac{\left[PgMA\right]}{{[PEGMEMA}_{300}]}\right)}_{poly}=\frac{\left[PgMA]\;(r_{1}[PgMA]+{\;[PEGMEMA}_{300}]\;\right)}{{[PEGMEMA}_{300}]([PgMA]+r_{2}[{PEGMEMA}_{300}]\;)}$$

**Figure 4:**
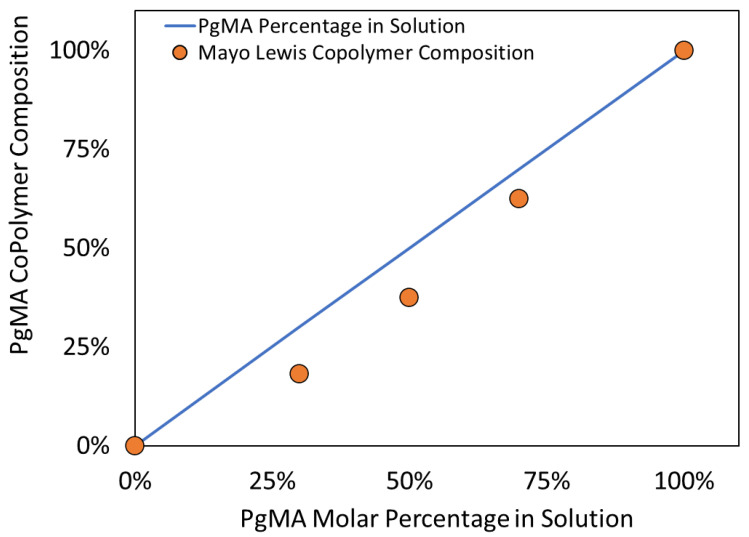
Composition of PgMA-*co*-PEGMEMA_300_ as calculated using the Mayo–Lewis equation using homo-polymerization rates from the gravimetric analysis.

Using the molar ratio of PgMA to PEGMEMA_300_, the molar composition can be calculated. The results in Figure 4 suggest that an increased rate of PEGMEMA_300_ polymerization leads to reduced PgMA incorporation at intermediate solution compositions.

#### 3.2.3. Water-Permeability Analysis of ATRP Modified Membranes

**Figure 5:**
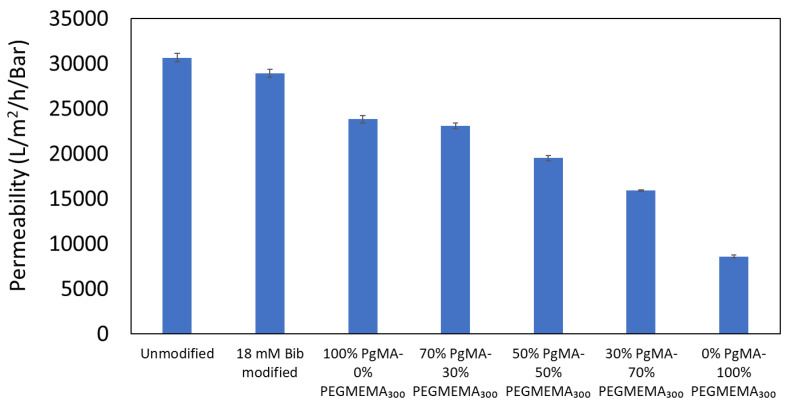
Permeability of PgMA-*co*-PEGMEMA_300_-modified membranes. Error bars represent the standard deviation of at least three measurements.

Pure water-permeability measurements are typically performed to ascertain the impact of polymerization on permeability due to the reduction in membrane pore size and porosity. Figure 5 shows that the membrane permeability decreases as the percentage of PEGMEMA_300_ in the polymerization solution increases. There is a 25% reduction in permeability from the unmodified membrane to the 100% PgMA membrane and a 72% reduction for the 100% PEGMEMA_300_ membrane, which is consistent with the DG_poly_ results.

Previous studies have shown reduced permeability after ATRP modification of regenerated cellulose membranes. Bhut et al. [23] performed ATRP of dimethylaminoethyl methacrylate from 1 µm pore diameter RC membranes and observed a permeability reduction of 41% after 12 h of polymerization. Wang et al. [24] performed ATRP of glycidyl methacrylate from the same membranes and reported a permeability reduction of 33% after 21 h of polymerization. In this study, modified membranes showed decreases in permeability with an increasing PEGMEMA_300_ percentage in the copolymer. Permeability was reduced from 28% to 72% as the percentage of PEGMEMA_300_ in the reaction solution increased from 0 to 100%. Coinciding with the reduced permeability was an increase in the DG_poly_ from ~61% to ~348%, going from 100% PgMA to 100% PEGMEMA_300_. These DG_poly_ values are high—for example, Bhut et al. [25] reported values from 9.5 to 22% for membranes grafted with 2-(methacryloyloxy)-ethyl]trimethylammonium chloride.

Because the permeability and DG_poly_ depend on the specific ATRP conditions, monomers, ATRP time, and test solvent, the exact reduction in the permeability and increase in DG_poly_ will vary for each case. In this study, incorporating PEGMEMA_300_ monomer greatly influenced the permeability and DG_poly_ due to differences in hydrophilicity between it and PgMA. PEGMEMA_300_ is known to swell in water, while PgMA is hydrophobic and collapses in the presence of water [26,27]. Since the copolymer is anchored covalently from the pore surface of regenerated cellulose membranes, polymer chains containing a high percentage of PEGMEMA_300_ will swell in the presence of water, causing a more significant reduction in permeability than chains containing a high percentage of PgMA that collapse in water.

The ATRP solvents also contributed to the high DG_poly_ and permeability loss observed in this study. DMSO and water, in particular, have been noted for their ability to increase the rate of polymerization in ATRP. This acceleration is attributed to two effects: (i) increases in the equilibrium (K_ATRP_) and activation rate (K_act_) and (ii) a decrease in the dissociation rate of the halide from the deactivator complex, resulting in more radicals and, therefore a higher rate of polymerization (k_p_) [28]. In a study performed by Braunecker et al. [29] that characterized solvent effects on ATRP, DMSO had the third-highest K_ATRP_ value, and water had the highest K_ATRP_ value out of 11 solvents commonly used. Huang et al. [30] polymerized 2-hydroxyethyl methacrylate from gold surfaces using ATRP. They found that polymerization in aqueous media resulted in polymer films with a thickness of 700 nm, whereas neat monomer polymerization resulted in films of only 6 nm. In our preliminary studies, methanol and acetone were tested as ATRP solvents, but they resulted in either yellow/greenish precipitation on the membrane surface or poor polymerization, as evidenced by ATR-FTIR. DMSO/H_2_O solvent enhanced PgMA solubility and qualitatively appeared to accelerate the polymerization rate based on the ATR-FTIR spectra (Figure S2 in Supplementary Materials).

### 3.3. Characterization of Clicked Membranes

ATR-FTIR Characterization of Clicked Membranes

In most studies, PgMA is polymerized after protection with a trimethylsilyl group because the acetylene moiety is known to interfere with polymerization via radical addition to the acetylene groups [20,31,32]. Since no protection was used in this case, it was essential to demonstrate that PgMA acetylene groups remained available for the click reaction. Clicked membranes were characterized by ATR-FTIR spectroscopy and gravimetric analysis.

**Figure 6:**
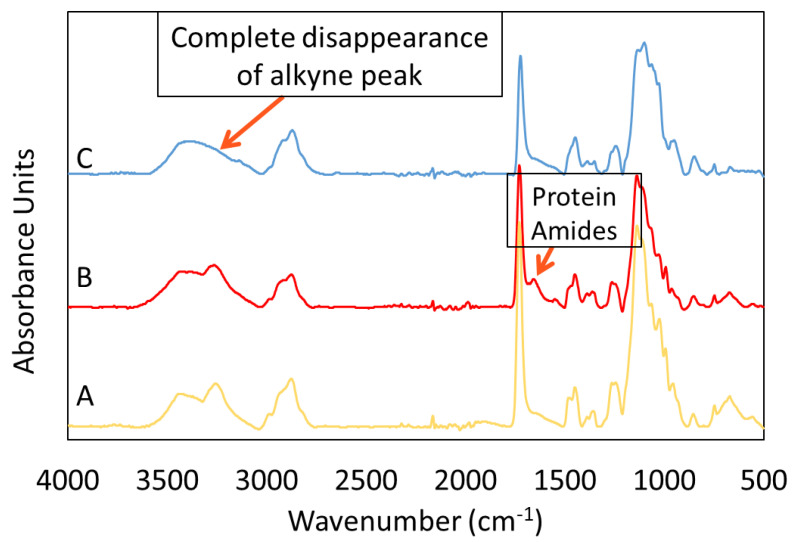
ATR-FTIR of modified membranes: (A) 30% PgMA-*co*-70% PEGMEMA_300_ membrane after 5 h ATRP modification; (B) 30% PgMA-*co*-70% PEGMEMA_300_, 5 h ATRP, clicked with azide-functionalized-Protein A; (C) 30% PgMA-*co*-70% PEGMEMA_300_, 5 h ATRP, clicked with azido-propanol.

Figure 6 shows ATR-FTIR spectra for 30% PgMA-*co*-70% PEGMEMA_300_ membranes clicked with azido-propanol and azide-functionalized Protein A and the spectrum for membranes prior to the click reactions. For azido-propanol-clicked membranes, a successful reaction is supported by the complete disappearance of the alkyne peak at 3245 cm^−1^. For membranes clicked with azide-functionalized Protein A, a successful reaction is supported by the appearance of amide stretching bands at 1644 cm^−1^ and 1536 cm^−1^. The alkyne peak at 3245 cm^−1^ is diminished but remains after the click reaction with alkyne-conjugated Protein A, which is likely due to the steric hindrance of large Protein A molecules and lower conversion compared to the azido–propanol click reaction.

From the literature, it is known that the ligand structure can have a significant impact on the click reaction. Therefore, a screening study was performed to test the effectiveness of different CuBr_2_-ligand catalyst complexes on the click reaction using azido-propanol. TPMA, THPTA, and PMDETA were evaluated in a 1:1 ratio with CuBr_2_. Membranes clicked using THPTA and PMDETA catalyst ligands had no peak at 3245 cm^−1^ in the ATR-FTIR spectra, indicating complete conversion of alkyne groups, whereas modification using TPMA as the catalyst ligand showed this peak. A recent study [17] noted that aliphatic ligands have much faster rates of click reaction than pyridine-based ligands. The study found that click reactions using PMDETA ligand was 230 times faster than reactions using no ligand. Comparatively, TPMA, a pyridine-based ligand, resulted in a reaction rate that was only 1.7 times higher than the case with no ligand. Aliphatic ligands accelerate the formation of a pi complex with Cu(I) in solution due to the increased basicity and ionization potential of aliphatic amines compared to pyridines. Similarly, tris(heterocyclomethyl)amines, like THPTA, utilize triazoles to stabilize Cu(I), which leads to better catalyst solubility and acceleration of the click reaction [17]. THPTA was selected for subsequent membrane synthesis.

### 3.4. Performance of Protein A Membranes

#### 3.4.1. Static Binding Capacity (SBC) Results

**Figure 7:**
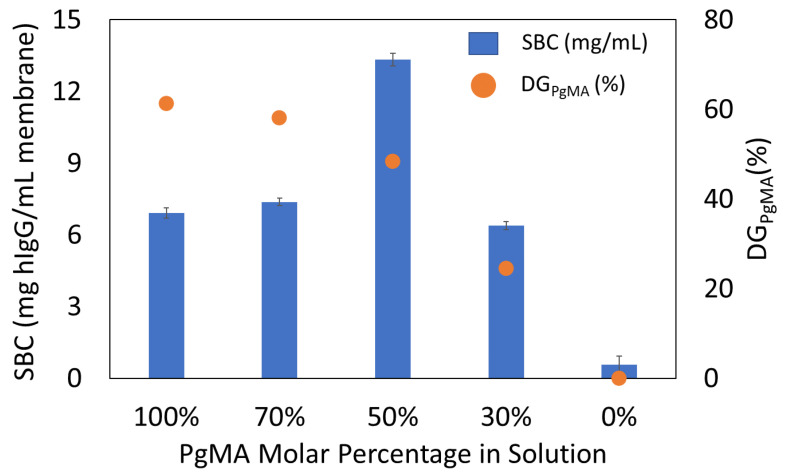
Static binding capacity of clicked Protein A membranes as a function of monomer molar percentages in the ATRP reaction solution.

The Protein A click reaction was performed on membranes modified with different PgMA-*co*-PEGMEMA_300_ copolymers using reaction solutions with PgMA molar percentages ranging from 0 to 100%. Following the click reaction, SBC measurements were conducted at 5 mg/mL hIgG. The resulting SBC values shown in Figure 7 highlight important factors influencing hIgG binding capacity. On the secondary y-axis, the DG_PgMA_ is graphed to show the correlation between SBC and the amount of PgMA grafted in copolymers.

Notably, the optimal binding polymer composition may not always occur at a 50% composition of the co-monomers. For example, in a study by Saha et al. [20], azidopropyl methacrylate-*co*-poly(ethylene glycol) methyl ether methacrylate (PEGMEMA_300_) polymer chains were grafted using surface-initiated ATRP from flat gold substrates. The polymer chains were then click-reacted with an alkyne-containing macromolecule (poly(ethylene glycol) methyl ether, M_n_ = 5000 kDa). The optimal condition was found at 75 mol % PEGMEMA_300_ in the copolymer chains [20].

In this study, the highest SBC occurred at 50% PgMA-*co*-50% PEGMEMA_300_ (Figure 7), and the effect of copolymer composition on static binding capacity can be explained in terms of the physicochemical properties of the copolymers. At a low PgMA percentage, the mass of PgMA incorporated in the copolymer was small. The copolymer is rich in PEGMEMA_300_, which increases swelling (as evidenced by previously discussed permeability studies). In turn, this increases Protein A ligand access to alkyne groups. However, the increased membrane thickness at lower PgMA percentages negatively affects the SBC, as it is calculated on a membrane volume basis. At higher PgMA percentages, the higher number of alkyne groups combined with lower membrane thickness positively affects SBC. However, reduced Protein A access to alkyne sites is expected because the hydrophobic polymers swell less in water. The balance among hydrophobicity/hydrophilicity, the incorporated amounts of PgMA and PEGMEMA_300_ spacer, and the membrane thickness leads to the 50% PgMA-*co*-PEGMEMA_300_ case being optimal for the static binding of hIgG.

**Figure 8:**
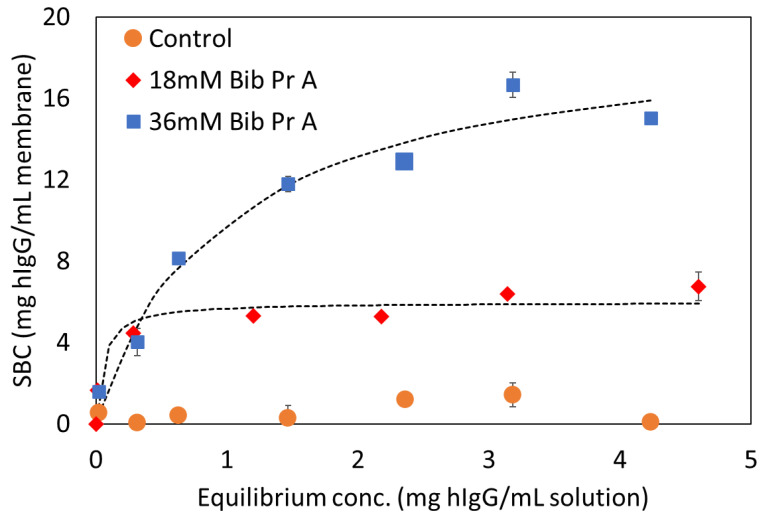
Static binding capacity measurements for Protein A membranes prepared using the 30 mol% PgMA ATRP formulation and 5 h polymerization. Curves represent the best fits to the Langmuir adsorption isotherm model.

In addition to copolymer composition, the initiator concentration used to activate membranes for ATRP also influences the SBC. Initiator concentrations of 18 and 36 mM Bib were tested using the 30 mol% PgMA ATRP formulation and a polymerization time of 5 h. Thermodynamic binding parameters, q_max_ and apparent K_d_, were calculated by fitting SBC isotherm data to the Langmuir model. Figure 8 compares the isotherms. The dashed curves represent the best fits to the Langmuir model. Table 2 presents the Langmuir fitting parameters, which suggest that the initiator density influences the interaction between Protein A and the hIgG ligand. A 2-fold increase from 18 to 36 mM Bib concentration led to an approximately 3.3 times increase in maximum binding capacity (q_max_). Similar behavior was reported by Bhut et al. [25]. Strong anion-exchange membranes produced by surface-initiated ATRP of 2-(methacryloyloxy)ethyl]trimethylammonium chloride showed a ~4 times increase in binding capacity of bovine serum albumin when initiator concentration was doubled from 9 to 18 mM Bib. One possible explanation for this is that, for membrane adsorbers prepared using initiator functionalization and subsequent AGET ATRP, the relationship between the initiator concentration in the solution and the initiator sites on cellulose membranes may not be linear.

For apparent K_d_, a significant difference was observed between membranes activated using 18 versus 36 mM Bib. The apparent K_d_ for membranes activated using 36 mM Bib is 18 times higher than those activated using 18 mM Bib. This suggests that a higher degree of grafting dramatically influences the affinity interactions between immobilized Protein A and hIgG. One explanation is that the Protein A ligand becomes more entangled at higher degrees of grafting, leading to a lower number of hIgG molecules bound per Protein A molecule. Similar observations have been made in the literature for other Protein A chromatography media. Penzol et al. [33] compared the IgG to Protein A binding ratios for dextran-agarose supports prepared with 6000 g/mol dextran versus 20,000 g/mol dextran. Dextran with 6000 g/mol showed a binding ratio of 2 IgG molecules to 1 Protein A, while dextran with 20,000 g/mol showed a binding ratio of 1.5 to 1. The authors credited increased ligand entanglement via higher-molecular-weight dextran as a source of steric hindrance that affected the binding ratio between IgG and immobilized Protein A [33]. The results from both these studies suggest that the degree of grafting can affect the stoichiometry between an immobilized ligand and its substrate, affecting the apparent K_d_ value.

#### 3.4.2. Dynamic Binding Capacity (DBC_10_) Results

DBC_10_ measurements were performed for membranes prepared using the 30 mol% PgMA ATRP formulation with membranes activated using 36 mM Bib initiator concentration. At least three runs were performed at each residence time (chromatograms shown in Figure S3 in Supplementary Materials). The resulting DBC_10_ was independent of the flow rate. At 30 s residence time, the average DBC_10_ was 5.17 ± 0.55 mg/mL, while at 300 s residence time, the average DBC_10_ was 4.50 ± 0.63 mg/mL.

## 4. Conclusions

This study presents a novel strategy for preparing Protein A affinity membranes by combining AGET ATRP of PgMA-*co*-PEGMEMA_300_ copolymers and Cu(I)-catalyzed alkyne–azide click chemistry. The influence of polymerization conditions on the Protein A affinity membrane performance was reported for the first time in the open literature. Copolymers grafted with PEGMEMA_300_ outperformed PgMA homopolymers due to the contributing factors of polymer hydrophobicity/hydrophilicity, the incorporated amounts of PgMA and PEGMEMA_300_ spacer (the composition), and the resulting membrane thickness after copolymerization. While increased PEGMEMA_300_ increases the swelling and the access of Protein A to the alkyne monomers, the high degree of grafting at higher PEGMEMA_300_ leads to increased membrane thickness, negatively affecting the SBC value. The 50% PgMA-*co*-PEGMEMA_300_ case provided the optimal SBC of hIgG for the polymerization conditions presented in this study. However, a different ATRP solvent system with a different co-monomer spacer may lead to different copolymer composition and swelling characteristics that would affect the final performance of the Protein A membranes.

Another parameter affecting Protein A membrane performance is the initiator concentration. Utilizing the same grafted copolymer composition at different initiator concentrations, uptake isotherms revealed that q_max_ and apparent K_d_ were affected by initiator concentration in different ways. Higher initiator concentration produced membranes with higher q_max_ but lower apparent K_d_. Finally, for membranes showing the highest q_max_, DBC_10_ values were measured and found to be independent of flow rate, suggesting that convective transport is maintained in membrane pores using this polymer grafting strategy.

The novel approach presented here is an advancement in the field of Protein A chromatography, as it shows that optimization of polymerization conditions for affinity membranes, although understudied in the literature, is an essential tool for tuning membrane adsorber performance. The approach and findings demonstrated here can be extended to developing other chromatography supports that require the immobilization of protein, peptide, and synthetic ligands.

## Figures and Tables

**Table 1:** Molecular weight increases after azido-butyric acid conjugation to Protein A at an azidobutyric acid NHS ester to Protein A molar ratio of 15 to 1 in solution. Values are the average and standard deviation of the peak for two different measurements.

MW/Charge (g/mol)	Control Protein A (g/mol)	Azide-Conjugated Protein A (g/mol)	Increase in Molecular Weight (g/mol)	Number of Azide Groups Added
M/Z	46,401 ± 48	47,836 ± 93	1435 ± 45	12.9 ± 0.4
M/2Z	23,099 ± 27	23,885 ± 14	785 ± 42	14.2 ± 0.8 *

* Calculated by multiplying the increase in molecular weight by 2 to account for the double charge.

**Table 2:** Fitted Langmuir parameters from static binding capacity experiments. q_max_ is the maximum binding capacity, and K_d_ represents the apparent dissociation equilibrium constant.

Langmuir Parameters	36 mM Bib, 30% PgMA-*co*-70% PEGMEMA_300_	18 mM Bib, 30% PgMA-*co*-70% PEGMEMA_300_
q_max_ (mg/mL)	19.60 ± 1.88	5.98 ± 0.37
Apparent K_d_ (mg/mL)	9.9 × 10^−1^ ± 2.9 × 10^−1^	5.4 × 10^−2^ ± 3.5 × 10^−2^

## Data Availability

The data presented in this study are available on request from the corresponding author.

## Supplementary Materials

The following supporting information can be downloaded at https://www.mdpi.com/article/10.3390/polym16020239/s1, Figure S1: Percentage increase in membrane thickness as a function of PgMA-PEGMEMA_300_ solution composition; Figure S2: ATR-FTIR of PgMA-*co*-PEGMEMA_300_ modified membranes prepared using different solvents; Figure S3: bind-and-elute chromatograms of 30% PgMA-*co*-PEGMEMA_300_-modified membranes prepared using 36 mM Bib and 5 h ATRP.
