# Supplementary material for: Preparation of Protein A Membranes Using Propargyl Methacrylate-Based Copolymers and Copper-Catalyzed Alkyne–Azide Click Chemistry
Source: Polymers (Basel). 2024 Jan 15;16(2):239. doi: 10.3390/polym16020239 (PMC10819539; doi:10.3390/polym16020239)
Supplement: Supplementary file 1 [file polymers-16-00239-s001.zip › polymers-2776701-supplementary.pdf]

polymers

MDPI

# Supplementary Materials: Preparation of Protein A Membranes Using Propargyl Methacrylate-based Copolymers and Copper-catalyzed Alkyne-Azide Click Chemistry

**Joshua Osuofa and Scott M. Husson**

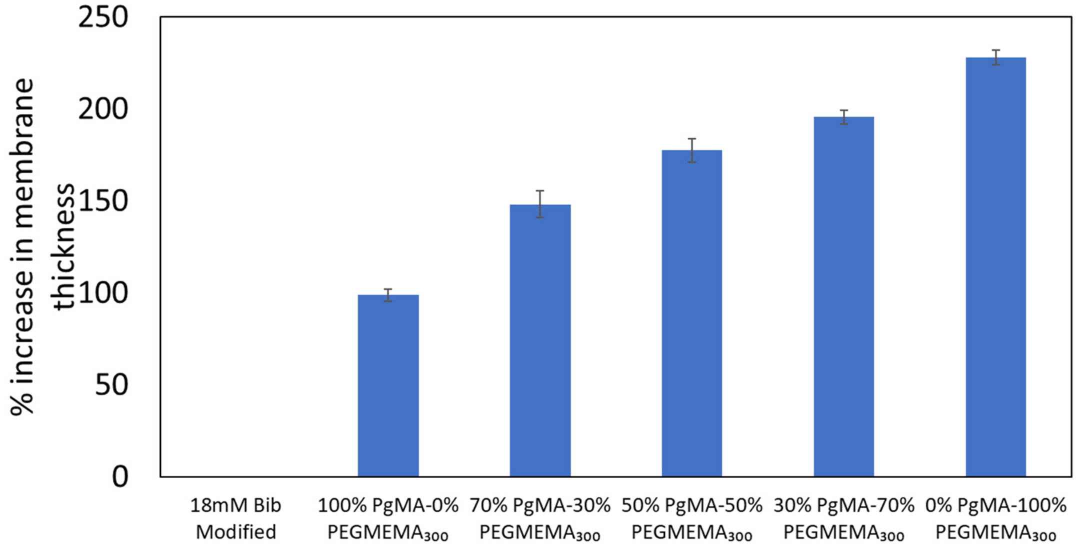


**Figure S1.** Percentage increase in membrane thickness as a function of PgMA-PEGMEMA300 solution composition. Error bars represent standard deviation of at least three measurements.

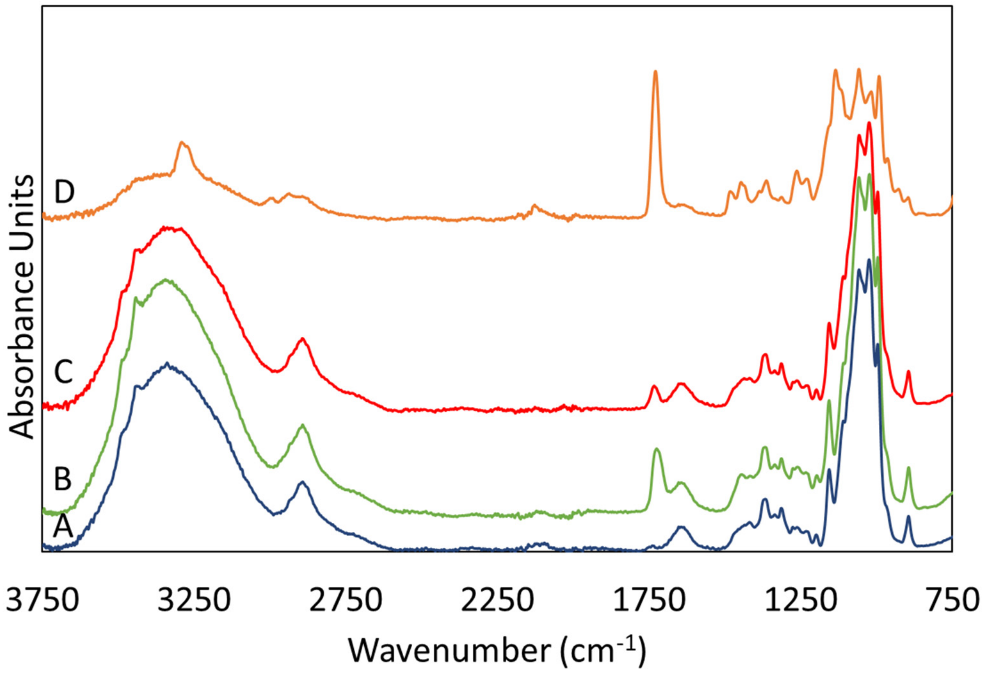


**Figure S2.** ATR-FTIR of PgMA-*co*-PEGMEMA300 modified membranes prepared using different solvents: (A) unmodified membranes, (B) methanol, (C) acetone, (D) DMSO/$H_2O$.

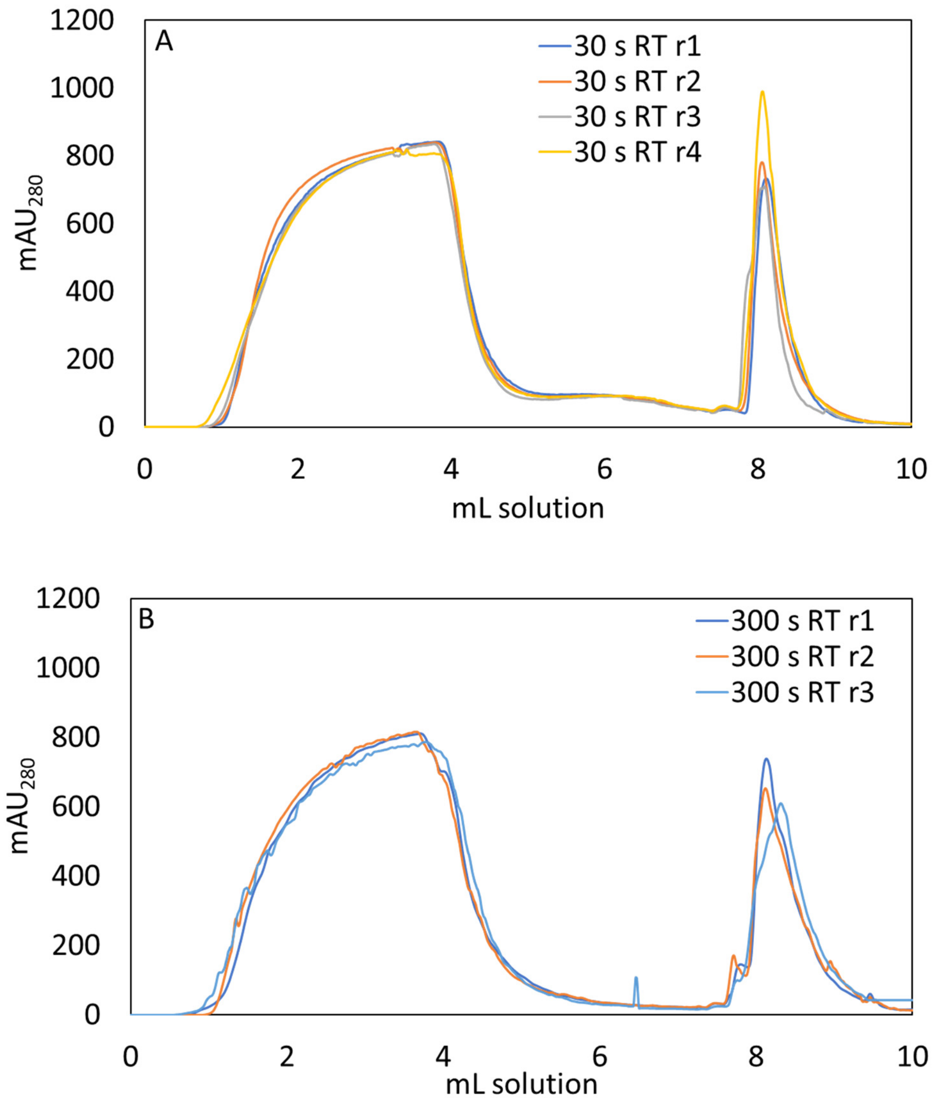


**Figure S3.** Bind-and-elute chromatograms of 30% PgMA-*co*-PEGMEMA$_{300}$ modified membranes prepared using 36 mM Bib and 5 h ATRP. (A) 30 second residence time. (B) 300 second residence time.
